# Burst-pause criterion derivation for drinkometer measurements of ingestive behavior

**DOI:** 10.1016/j.mex.2022.101726

**Published:** 2022-05-11

**Authors:** Michele Serra, Bálint File, Daniela Alceste, Ivana Raguz, Daniel Gero, Andreas Thalheimer, Jeannette Widmer, Aiman Ismaeil, Robert E. Steinert, Alan C. Spector, Marco Bueter

**Affiliations:** 1 Department of Surgery and Transplantation, University Hospital Zurich, Switzerland; 2 Faculty of Information Technology and Bionics, Pázmány Péter Catholic University, Budapest, Hungary; 3 Wigner Research Centre for Physics, Budapest, Hungary; 4 Department of Psychology and Program in Neuroscience, Florida State University, Tallahassee, FL, USA

**Keywords:** Drinkometer, Ingestive behavior, Burst-pause criterion, Weight loss, Obesity

## Abstract

The drinkometer is a promising device for the study of ingestive behavior of liquid meals in humans. It can be used to investigate behavior in different target populations. However, ingestive behavior has a great variability across study participants. Therefore, a new analytical approach is required for the extraction and analysis of drinkometer-derived data that could account for this variability. We developed an optimized protocol to predict an optimal burst-pause criterion (PC) for the extraction of PC-dependent microstructural parameters of ingestive behavior. These describe the microstructure of bursts, while PC-independent parameters describe the microstructure of sucks. Therefore, a PC is required to analyze separately two physiologically different parts of behavior. To accomplish this burst-pause criterion derivation (BPCD), a Gaussian Mixture Model (GMM) was built for estimation of two probability density functions (PDFs). These model the distribution of inter-suck intervals (ISIs) and inter-burst intervals (IBIs), respectively. The PC is defined at the intersection point of the two density functions. A Kaplan-Meier (KM) survival analysis was performed for post-hoc verification of the fit of the predicted optimal PC to the ISI distribution. In this protocol paper, we present a walkthrough of the data analysis of drinkometer-derived data for the measurement of microstructure of ingestive behavior based on previous results published by our group [1].

• Standardization of the burst-pause criterion derivation for drinkometer measurements of ingestive behavior.
• All codes are publicly available in a repository.
• The method can be easily adapted to studies with larger sample size or more than one study stimulus.

## Graphical abstract

**Figure:**
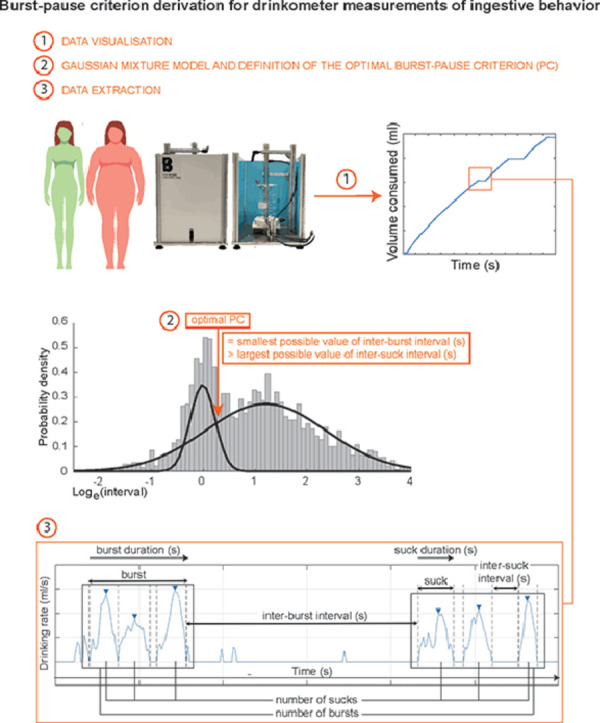

Specifications table

**Table:** 

Subject Area:	Medicine and Dentistry
More specific subject area:	Ingestive behavior
Method name:	Burst-Pause Criterion Derivation (BPCD)
Name and reference of original method:	Gero, D., File, B., Justiz, J., Steinert, R. E., Frick, L., Spector, A. C., & Bueter, M. (2019). Drinking microstructure in humans: a proof-of-concept study of a novel drinkometer in healthy adults. Appetite, 133, 47-60. The original method was published by our group in 2019 in a research paper describing the study of ingestive behavior in humans with a drinkometer device. The present protocol paper describes how to derive the burst-pause criterion from the drinkometer-derived data for the extraction of the burst-pause-criterion-dependent microstructural parameters of ingestive behavior.
Resource availability:	Softwares and packages: MATLAB (version R2020a), https://uk.mathworks.com/products/matlab.html[2] R (version 4.0.3), https://www.r-project.org[3] RStudio (version 1.3.1093), https://www.rstudio.com[4] *survminer* R package, https://cran.r-project.org/web/packages/survminer/readme/README.html[5] Source code repository: https://github.com/micheleserra/bueter.lab/tree/Drinkometer
Ethics:	The study was carried out according to the Declaration of Helsinki. Ethical approval was received from the Cantonal Ethical Committee of Zurich (BASEC-Nr. 2017-00756). Informed consent was obtained from all participants.
Trial registration:	The protocol was registered at ClinicalTrials.gov with Identifier NCT04933305.

For complete details on the presented data, and on the use and execution of this protocol, please refer to Alceste at al. [1].

## Description of the method

In this protocol paper, we present a walkthrough of the data analysis of drinkometer-derived data for the measurement of microstructure of ingestive behavior based on previous results published by our group [1]. The objective of this protocol was to provide a standardized methodology for extraction and processing of drinkometer-derived data. This would ensure a reproducible and data-driven analysis for the study of human ingestive behavior during liquid meals recorded with a drinkometer. Further detailed information on the general interest and importance of this topic and method is reported in the background information section.

As recently described by our group [1], human ingestive behavior is quantified with two sets of parameters: macrostructural and microstructural. The set of macrostructural parameters is the scaffold which gives a shape to the behavior, while the set of microstructural parameters describes the fine-grained complexity of individual behavior. The parameters comprised in this set are based on sucks and bursts, the latter being defined by the former with a burst-pause criterion (PC) interval. Bursts are indeed clusters of sucks. They are physiologically different from sucks in relation to the length of intervals between them. The physiological mechanism regulating the length of these intervals remain unknown. The identity of an interval as an inter-suck interval (ISI) or as inter-burst interval (IBI) is declared according to the distribution of all recorded intervals of a meal test. By observing the distribution of all intervals, it is possible to define a probability for each interval to be an ISI or an IBI. The PC is defined as the intersection point of two probability density functions (PDFs) modelled on the distribution of all intervals. The PC (the intersection point of the two density functions) represents the cutoff of the area under the two curves. On the left of the intersection point, it is more probable that a value represents an ISI (ISI < PC). On the right of the intersection point, it is more probable that a value represents an IBI (IBI ≥ PC).

### Required softwares and packages for data processing and analysis

Drinkometer-derived data were processed as previously described [6]. Briefly, raw data were extracted and pre-processed with MATLAB (version R2020a, Mathworks, Natick, MA) for (1) estimation of the burst-pause criterion (PC)-dependent microstructural parameters, and (2) calculation of the mean value of each macro- and microstructural parameter for each test session. Macro- and microstructural parameters are described in Alceste et al. [1]. Data analysis of pre-processed drinkometer-derived data was performed using R (version 4.0.3) [3] via RStudio (version 1.3.1093) [4]. The overall probability of inter-suck intervals (ISIs) derived with different PCs was analyzed with a Kaplan-Meier (KM) survival analysis. KM survival curves were generated with *survminer* R package [5].

#### Data extraction from raw drinkometer-derived data

Drinkometer measurements produce *.info, .tdms*, and *.tdms_index* files that need to be processed for further statistical analysis. We wrote six independent, but sequential codes:

1. *plot_tdms.m* for visualizing raw consumed volumes and identifying eventual disturbances in the measurements. The visualization of raw volumes is discussed further below.
2. *create_ISI_table.m* for extracting all ISIs of all participants.
3. *ISI_histograms.Rmd* for visualization of the frequency density of the ISIs of each participant.
4. *GM_for_IBI.m* for identifying the PCs based on a Gaussian Mixture model (GMM), and for plotting the probability density functions (PDFs) of the cumulative ISIs.
5. *KM_survival_analysis.Rmd* for comparing the time-to-event distribution of ISI probability between ISIs derived with the optimal PC and ISIs derived with arbitrary PCs.
6. *create_all_the_tables.m* for extracting the data by applying the estimated optimal PC.

STEP 0

1. Open MATLAB and add the folder with the drinkometer-derived data to the path.

STEP 1: Visualization of raw volumes

**Fig. 1:**
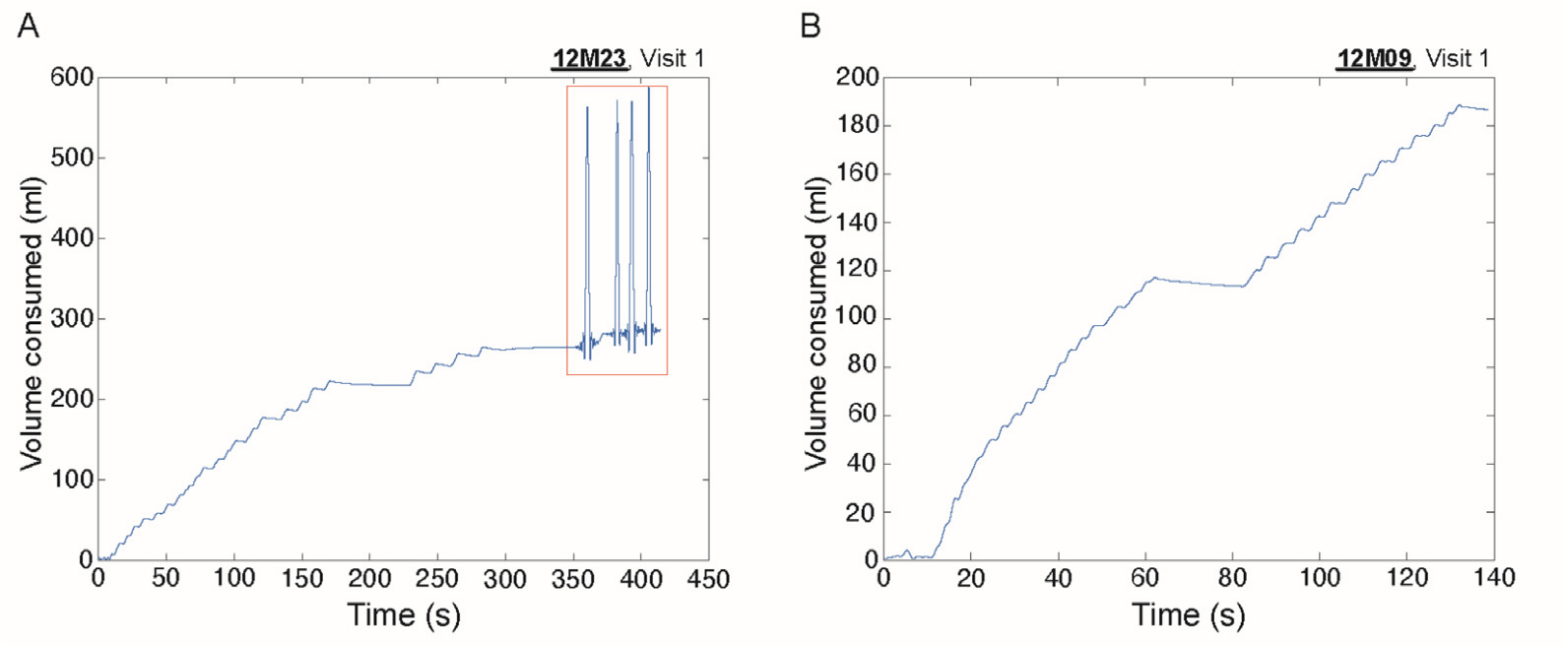
Raw volumes of drinkometer measurements. (A) Consumed volume of participant 12M23 at visit 1 as a function of time. Interferences were observed at the end of the recording (orange box). This and all other drinkometer measurements of this study participant were excluded from the data analysis. (B) Consumed volume of participant 12M09 at visit 1 as a function of time. No absence of signal or interferences were observed. This and all other drinkometer measurements of this study participant were included in the data analysis.

1. Open the code *plot_tdms.m*.
2. Set the sampling frequency of the signal (Fs) – suggested 1000 (Hz).
3. Set the cutoff frequency (FOI) for low-pass filtering – suggested 0.5 (Hz).
4. Set *plot_filtered_volume* to 1 to visualize the raw volumes with a cutoff frequency of 0.5 Hz.
5. Run the script for the selected measurements. The script needs three files for each measurement: *.info*, .*tdms*, and *.tdms_index*. These files are automatically generated as output of the drinkometer measurements.
  a. The *.info* file contains information about the operational settings of the measurements and the reference values of cup weights, cup internal and external diameters, metal and rubber tube lengths, metal and rubber tube internal and external diameters, and calibration of scales, ultrasound sensors and temperature sensors.
  b. The *.tdms* file contains the measurement data.
  c. The *.tdms_index* file contains information about the drinkometer conditions throughout the measurement.
6. Open the newly generated figures and exclude measurements with interferences (Fig. 1A) and continue the analysis only with complete and clean measurements (Fig. 1B). In Fig. 1A, within the orange box, it is possible to see how interferences appeared in the visualization of raw volumes of the study participant 12M23. This participant was excluded from the analysis.

STEP 2: Visualization of inter-suck intervals

The following settings were optimized for our device. It is possible that for the same device in other environmental conditions, the settings would need to be adjusted.

**Fig. 2:**
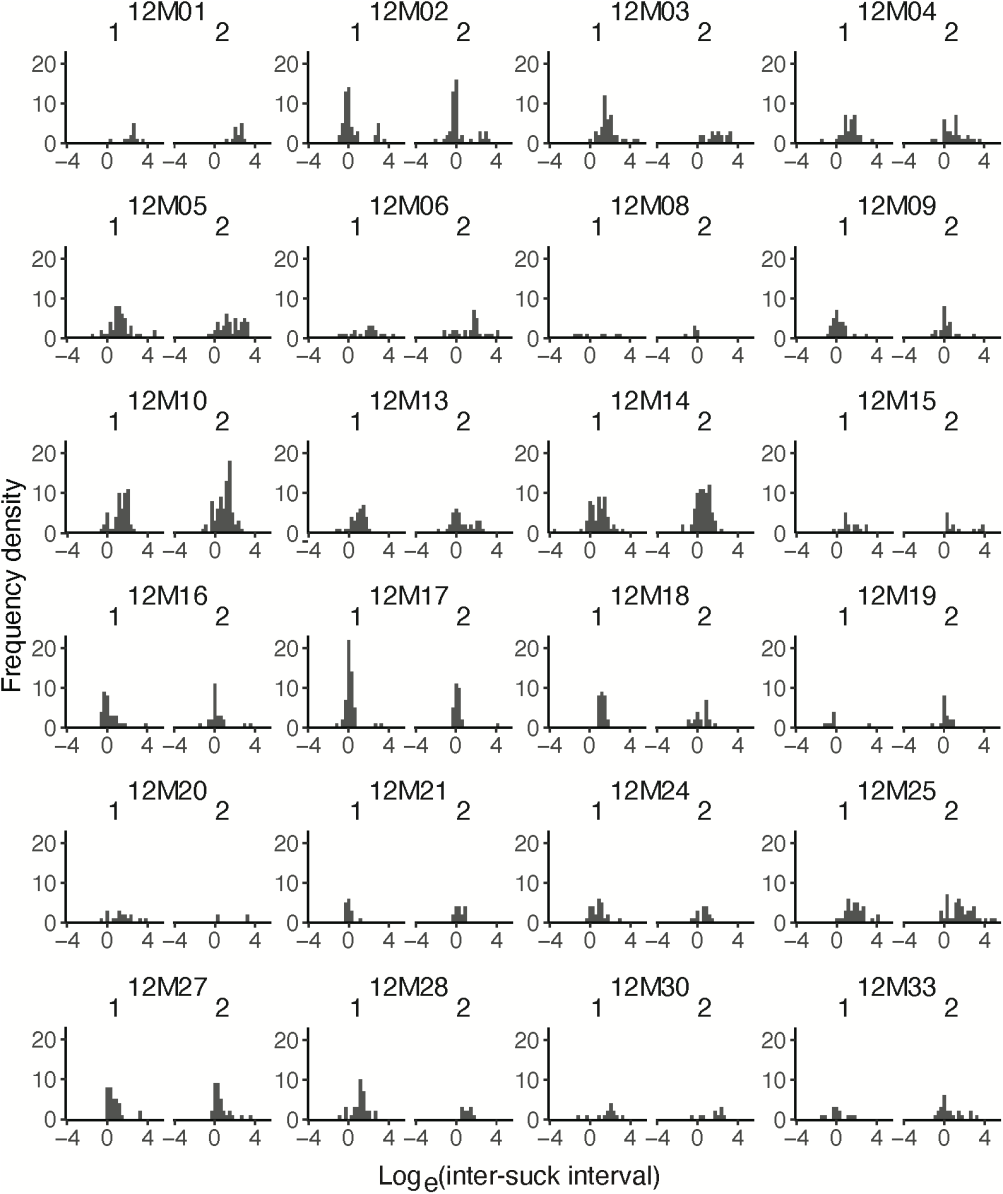
Frequency density of intervals, expressed in logarithmic scale, for each participant. The density distribution is shown separately for visit 1, on the left, and visit 2, on the right.

1. Open the code *create_ISI_table.m*.
2. Set the sampling frequency of the signal (Fs) – suggested 1000 (Hz).
3. Set the cutoff frequency (FOI) for filtering – suggested 0.5 (Hz).
4. Set *plot_filtered_volume* to 1 to visualize the raw volumes with a cutoff frequency of 0.5 Hz.
5. Set which speed signal in absolute value is considered as an artefact above the threshold – suggested 20 (ml/s). The detected interval is deleted and interpolated linearly.
6. Set the minimal amplitude of sucks – suggested 0.5 (ml/s). Below this threshold, the speed is set to 0. However, this value is highly dependent from the signal-to-noise ratio. Preliminary measurements are advised to assess the operational conditions of the device.
7. Set the minimal interval between peaks of sucks to prevent detecting noise as sucks – suggested 1 (s).
8. Set the minimal suck size – suggested 1 (ml). Below this threshold, the sucks are considered as noise and are not included in the analysis.
9. Run the script for the selected measurements. The script needs three files for each measurement: *.info, .tdms*, and *.tdms_index*.
10. Open the code *ISI_histograms.Rmd*.
11. Set the directories as indicated in the script.
12. Run the script for the newly created *.csv* output of *create_ISI_table.m*.
13. Open the newly generated figure to observe the frequency density of ISIs for each participant (Fig. 2).

This output provides a graphical representation of the between-subject variability of the ingestive behavior of the study population. In theory, for each participant, an optimal PC should be defined for the extraction of PC-dependent microstructural parameters. Indeed, the density distributions of ISIs and IBIs differ between subjects, which might be a limitation of between-subject comparisons of PC-dependent microstructural parameters when these are extracted for all study participants with the same PC. Bootstrapping would be a suitable method for the estimation of the sampling distribution of ISIs and IBIs for each study participant. However, this method would require that both ISIs and IBIs have been sufficiently recorded in the measurements. This is not always the case because some measurements might be too short to allow recording of even one IBI. Considering that BPCD requires an intersection point between the Gaussian models of ISIs and IBIs, for short measurements would not be possible to derive a PC after estimating the sampling distribution by bootstrapping. Nevertheless, it is possible to build the GMM on the ISIs and IBIs distribution of the entire study population.

*STEP 3: Gaussian mixture model*

**Fig. 3:**
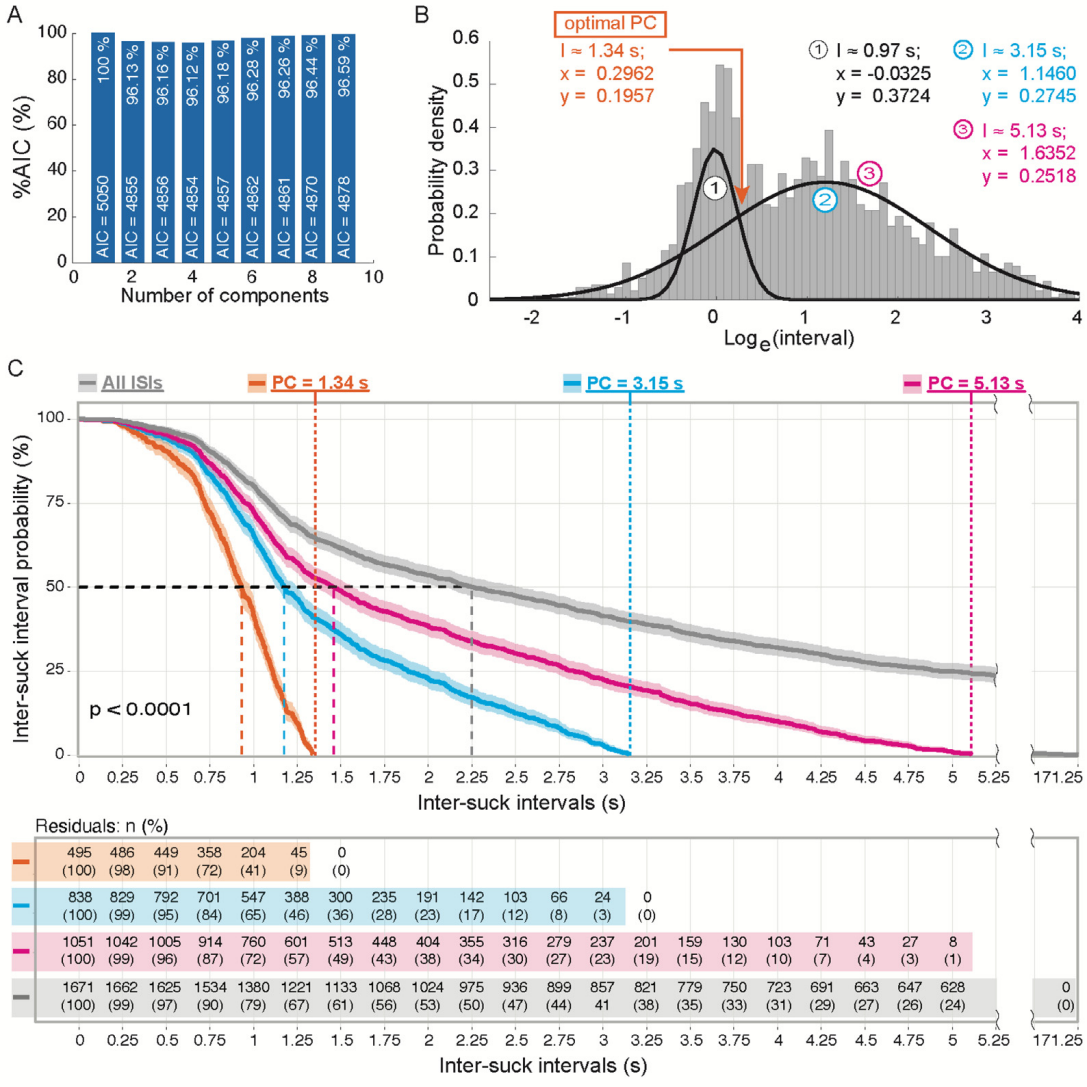
Analysis of the optimal burst-pause criterion (PC). (**A**) Number of components that minimize the information loss in the Gaussian Mixture Model (GMM) as estimated with the Akaike information criterion (AIC). The AIC value for each number of components is expressed as relative percentage in comparison with the AIC value of the single component (AIC = 5050). (**B**): Probability density functions of log_e_ transformed intervals for the identification of the optimal PC. The curve on the left describes the distribution of the ISIs, while the curve on the right describes the distribution of the estimated inter-burst intervals (IBIs). The peak of the density curve on the left (1) represents the most frequent value of ISIs (value (log*_e_*) = –0.0325; probability density = 0.3724). The peak of the density curve on the right (2) represents most frequent value of IBIs (value (log_e_) = 1.1460; probability density = 0.2745). The arrow points to the local minimum, i.e., the optimal PC for the extraction of microstructural PC-dependent parameters. Interval values shown on the top right are back-transformed from the respective logarithmic values (x). (**C**) Top, Kaplan-Meier survival curves were plotted for the probability of ISIs derived with three different PCs (1.34 s, 3.15 s, and 5.13 s) and all ISIs (grey curve). Bottom, residuals of the ISIs are reported with an interval of 0.25 s. **Abbreviations**: AIC, Akaike information criterion; I, interval; ISI, inter-suck interval; PC, optimal burst-pause criterion; PDF, probability density function; x = logarithmic value (log_e_); y = probability density.

1. Open the code *GM_for_IBI.m*.
2. Set the parameters as previously described for *create_ISI_table.m*.
3. Run the script for the selected measurements. The script needs three files for each measurement: *.info, .tdms*, and *.tdms_index*.
4. Open the newly generated figures to analyze the output of the Akaike information criterion (AIC, Fig. 3A) and of the GMM (Fig. 3B).

When considering the drinkometer measurements of the entire study population, the AIC estimated that a GMM with two density functions, as expected, would have had the smallest loss of information and the greatest goodness of fit to the recorded data (Fig. 3A). Indeed, each of the two density functions should model the ISIs and the IBIs, respectively. A probability density function (PDF) was applied to calculate the optimal PC for processing of the recorded data, as previously reported [6]. At the intersection point of the two density functions, a PC of 1.34 s was predicted as optimal for the discrimination of ISIs and IBIs of the current study population (Fig. 3B).

**Fig. 4:**
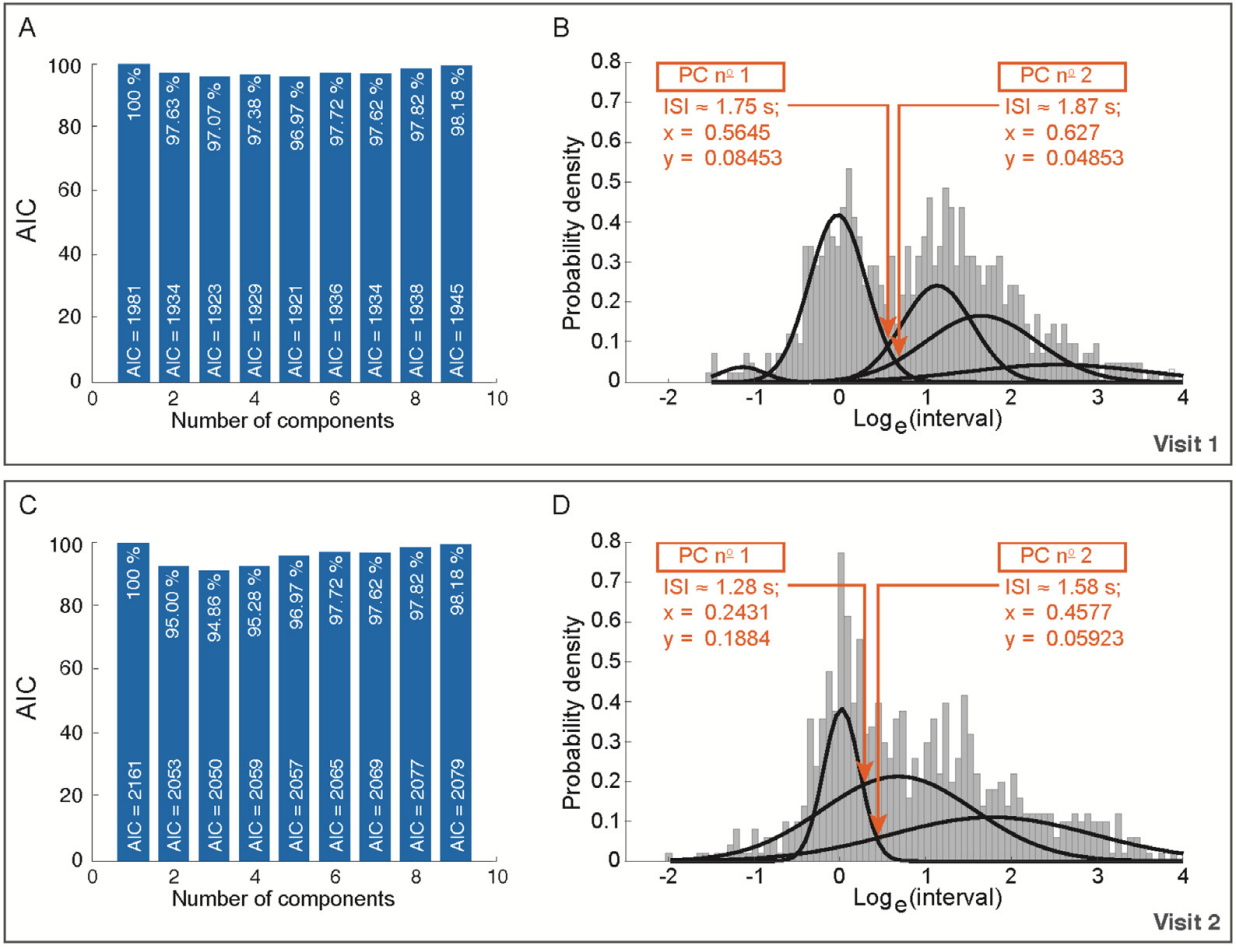
Analysis of the optimal burst-pause criterion (PC) of the two study visits. (**A**) Number of components that at visit 1 minimize the information loss in the Gaussian Mixture Model (GMM) as estimated with the Akaike information criterion (AIC). The AIC value for each number of components is expressed as relative percentage in comparison with the AIC value of the single component (AIC = 1981). (**B**) Probability density functions of log_e_ transformed intervals for the identification of the optimal PC at visit 1. The two arrows points to the local minimi, i.e., the two possible optimal PC for the extraction of microstructural PC-dependent parameters. (**C**) Number of components that at visit 2 minimize the information loss in the GMM as estimated with the AIC. The AIC value for each number of components is expressed as relative percentage in comparison with the AIC value of the single component (AIC = 2161). (**D**) Probability density functions of log_e_ transformed intervals for the identification of the optimal PC at visit 2. The two arrows points to the local minimi, i.e., the two possible optimal PC for the extraction of microstructural PC-dependent parameters.

Noteworthy, there might be an infinite number of PDFs. However, we are interested only in the two PDFs with the highest probabilities. These should represent the distribution of ISIs and IBIs, respectively. Modelling of two PDFs for the definition of a single PC was not possible when analyzing the interval distributions separately for the two study visits. Probably, not enough observations were included in the measurements. Indeed, the AIC estimated that a GMM with three density functions would have had the best fit to the data, and two possible optimal PCs were predicted for each model: (1) 1.74 s and 1.87 s, and (2) 1.28 s and 1.58 s for the intervals of visit 1 and visit 2, respectively (Fig. 4). This modelling limitation was dealt with by pooling together the observations of two study visits. Therefore, by increasing the numbers of observations, it was possible to have two sampling distributions which the two expected density functions could be modelled on. As mentioned above, even though this was not possible for the current data, bootstrapping might be a suitable statistical method to deal with non-normal sampling distributions of intervals.

*STEP 4: Post-hoc verification of the optimal burst-pause criterion*

1. Open the code *KM_survival_analysis.Rmd*.
2. Set the directories as indicated in the script. The data directory should contain the *.csv* output of *create_ISI_table.m*.
3. Set the optimal PC or any other arbitrary PC to be submitted to the KM survival analysis in the section “Selection of PC models” (ll. 37-49).
4. Run the script.
5. Open the newly generated figure to analyze the ISI probability of each recorded ISI according to each applied PC model and no PC model (Fig. 3C).

We performed a KM survival analysis of the overall probability of the total number of ISIs extracted with three different PCs (Fig. 3C): (1) the estimated optimal PC of 1.34 s (n = 495), (2) a PC of 3.15 s (n = 838), which, in the PDF model (Fig. 3B), represented the value of the second Gaussian curve with the highest probability, and (3) a PC of 5.13 s (n = 1051), which represented the first value of the second Gaussian curve for which there was minimal probability overlap with the first Gaussian curve.When an infinite PC was applied, n = 1671 ISIs were extracted. We compared these probabilities with a log-rank test. ISIs extracted with the optimal PC (1.34 s) demonstrated shorter overall time-to-event (p < 0.0001) compared to those derived from the PCs of 3.15 and 5.13 s (Fig. 3C). Based on a significance level of α = 0.05 (5 %), the null hypothesis of no difference in the time-to-event curves of the ISIs derived with the three considered PCs was rejected. Furthermore, the 50 % probability of the three survival curves appeared to divide the derived ISIs with different quotients of the respective PCs (Fig. 3C). With a PC of 1.3 s, a 50 % inter-suck interval probability was associated with ∼59 % of the observed ISIs (at 0.80 s), while, with a PC of 3.14 and 5.13 s, a 50 % inter-suck interval probability was associated with ∼36 % (at 1.12 s) and ∼28 % (at 1.44 s) of the observed ISIs, respectively. Consequently, a selection of intervals larger than the one corresponding to the intersection point for the definition of the PC might cause the erroneous classification of IBIs as ISIs. Indeed, considering a normal sampling distribution of ISIs, the 50 % inter-suck interval probability should be associated to approximately 50 % of the observed ISIs. Instead, this percentage increased parallelly to the increase of difference between the optimal PC and the alternatively selected interval.

STEP 5: Data extraction

1. Open the code *create_all_the_tables.m*.
2. Set the parameters as previously described for *create_ISI_table.m*.
3. Set the PC according to the results of the GMM and KM survival analysis.
4. Run the script for the selected measurements. The script needs three files for each measurement: *.info*, .*tdms*, and *.tdms_index*.
5. Open the newly generated tables to start the data analysis of macrostructural parameters, and PC-independent and PC-dependent microstructural parameters of human ingestive behavior for drinkometer-recorded liquid meals.

### Method validation

The AIC estimated that two density functions would have had the best fit to the data for explaining the recorded intervals (Fig. 3A). This is in accordance with our previously published work [6,7] but also conceptually aligned to experimental results on microstructure of licking behavior in animal models [8,9]. Indeed, it was previously reported that rodents exhibit three kinds of pauses in their licking behavior: inter-lick intervals, inter-burst intervals, and inter-cluster intervals, which are clusters of bursts separated by longer pauses [10]. While inter-lick intervals can be compared to ISIs of human subjects, there is a question whether the cluster/burst distinction is relevant to human sucking or whether it relates to differences in the topography of responses between the two species. For example, in rodents, the topography is licking and in humans it is sucking. When rodents lick, the tongue, although protruded, occasionally misses the drinking spout and this can lead to some of inter-lick intervals being considered inter-burst intervals [11]. Regardless, in the data presented in this protocol and in Alceste et al. [1], we were able to define at least two distributions accounting for ISIs and IBIs, respectively. We analyzed the data accordingly.

In a previous study [7], we reported that an optimal PC of 3 s poorly fitted the data (AIC = 13251.8, n = 126 measurements) [7], while, with the current study population (n = 48 measurements), we achieved an AIC of 4855 with the PC of 1.34 s. Considering that AIC scales with the sample size of the dataset and considering that a smaller AIC value is preferable to a larger one [12,13], this represents an advance in the analysis of drinkometer-derived data. Indeed, the inclusion of a single population of RYGB patients for the prediction of the optimal PC led to a model with a slightly better fit to the current data (4855/48 = 101.14) than the one we achieved in our previous work (13251.8/126 = 105.17). This suggests that the PC should be calculated separately for different populations. Future studies should test if the calculation of the PC for individual participants could provide an even better model. Individual PCs might provide the advantage to extract more accurate PC-dependent microstructural parameters. Unfortunately, the short duration of the measurements and the consequent limited number of intervals of our recordings prevented the application of a GMM for each study participant (Fig. 4). Therefore, we were not able to do this with the present data.

With the current approach and methodologies, it is difficult to assess the sensitivity and specificity of the predictive ability of different PCs for the prediction of ISIs and IBIs. In the present study, we performed a KM survival analysis to assess possible differences in ISI probability between the predicted optimal PC and two different arbitrary PCs . We could find a significant difference in their ISI probabilities. This was not only caused by the substantial difference given by the arbitrary length of the PCs but also by the distribution of the ISI probability within each PC. Indeed, for the non-optimal PCs of 3.15 s and 5.13 s, it appeared that the 50 % ISI probability occurred in the first third and quarter of the arbitrary PCs, respectively. While for the predicted optimal PC, the ISI probability was more equally distributed in the two halves of the PC. The sensitivity and specificity of an optimal PC determined with the current approach could, eventually, be compared against other arbitrary PCs by analyzing their true positive rate and false positive rate for the predicted ISIs and IBIs. However, the conceptual limitation to such an approach is that the physiological mechanism that controls ISIs and IBIs remains unknown. Consequently, these intervals represent methodological constructs for the analysis of ingestive behavior, where the components of a seemingly similar behavior are differentiated according to their sampling distribution.

The limitation of this approach is that the predicted optimal PC will, in this case [1], set the cutoff of ISI/IBI at suboptimal values for some of the participants. This is a limitation of this approach that need to be dealt with in future studies. Eventually, stricter rules in the inclusion criteria of the measurements into the data analysis might be necessary. Stricter criteria, however, require lager sample sizes, which we do not have yet, but that we are planning to recruit for further studies.

At the current state of the art, an optimal PC is considered to be at the intersection point of the two Gaussian probability density functions predicted by the GMM. However, it is difficult, if not virtually impossible, to define a PC that entirely separates distributions of ISIs and IBIs. Thus, considering also the novelty of this approach in humans, it is advisable to analyze the data using several arbitrary PCs to see if and how the nature of the outcomes of the experimental manipulations change.

## Additional information

### General background information

The characterization of licking microstructure in rodents during liquid meals has been proven a useful approach in the study of ingestive behavior and its neural controls [8,^10^,^11^,^14^,15]. A similar approach applied in humans is a promising methodology to investigate specific behavioral parameters underlying an alteration of appetite control [16]. Even though some authors have been investigating and developing devices such as universal eating monitors [17], a sipometer [18], and wearable devices [19], microstructure of human ingestive behavior has not been comprehensively analyzed in relation to body weight control.

Based on this theoretical framework, our group has recently developed a novel device, referred to as a drinkometer, which can be used for the analysis of the microstructure of human ingestive behavior. In a pilot study published in 2019, we reported that drinkometer-derived data could be employed to detect differences in microstructure within and between lean individuals, such as the size and number of sucks and their clustering in bursts. These differences were detected according to changes in concentration of the consumed sugar solution and to the fasting states of the study participants [6]. Consequently, the drinkometer is a promising research tool for the analysis of the mechanisms which control human ingestive behavior under diverse experimental conditions.

## Author contributions

Development of data-processing algorithm: BF, MS. Data acquisition: DA, IR. Data analysis: MS, DA. Drafting of the manuscript: MS, DA, MB. Critical revision and final approval of the manuscript: all authors.
